# Renoprotective effects of the ginger (*Zingiber officinale*) on Diabetic kidney disease, current knowledge and future direction: a systematic review of animal studies

**DOI:** 10.1186/s12906-022-03768-x

**Published:** 2022-11-11

**Authors:** Parisa Veisi, Meysam Zarezade, Helya Rostamkhani, Zohreh Ghoreishi

**Affiliations:** 1 Student Research Committee, Tabriz University of Medical Sciences, Tabriz, Iran; 2 Department of Clinical Nutrition, Faculty of Nutrition and Food Science, Tabriz University of Medical Sciences, Tabriz, Iran; 3 Nutrition Research Center, Faculty of Nutrition and Food Sciences, Tabriz University of Medical Sciences, Tabriz, Iran

**Keywords:** Ginger, Zingiber officinale, Diabetes, Hyperglycemia, Renal, Kidney

## Abstract

**Objective:**

Diabetic kidney disease affects approximately 40% of diabetic patients and is the leading cause of chronic kidney disease (CKD) worldwide. As a result, preventing renal complications in diabetic patients is critical. Ginger (*Zingiber Officinale Rosco*) is a popular spice and natral medicine. The present study was a systematic review focused on the existing evidence of the renoprotective effect of ginger extract on some features of diabetic kidney disease.

**Methods:**

The literature was searched in online databases such as PubMed, Scopus, EMBASE, ProQuest databases, and Google Scholar from inception to July 2022.

**Results:**

This review included 41 articles that met the eligibility criteria. Ginger supplementation was found to be associated with a significant decrease in blood glucose in 28 studies. Nine studies showed a significant reduction in malondialdehyde (MDA) after supplementation. Also, seventeen studies showed decreased serum levels of creatinine. Fifteen studies reported a decrease in total cholesterol (TC) and fourteen studies showed a lowered triglycerides (TG) concentrations. In twenty-six studies, ginger reduced renal injuries due to diabetes.

**Conclusion:**

Ginger may improve blood sugar indices, lipid profile, some inflammatory markers, oxidative stress, and pathologic injuries in diabetic kidney disease. However, future well-designed clinical trials and meta-analyses are required for a solid consensus.

## Introduction

Diabetic kidney disease (DKD) formerly known as diabetic nephropathy (DN), is a microvascular complication of diabetes, occurring in about one-third of people with diabetes [1–3]. The International Diabetes Federation estimates that the disease will increase from 463 million in 2019 to 700 million in 2045 [4]. DKD patients have a high incidence of cardiovascular morbidity and mortality [5]. The cause of the pathogenesis of DKD is multifactorial. Hyperglycemia is a key factor in the progression of pathologic alterations in the kidneys [6]. Similarly, dyslipidemia is a predictive factor in DKD progression [7]. In diabetes, increased oxidative stress plays a pivotal role in the development of DKD [8]. Also, inflammation has a crucial role in the onset and progression of DKD [9]. Today, the use of nutrition therapies and nutritional supplements along with treatment strategies to control the risk factors for cardiovascular disease in patients, as well as those with kidney diseases, has received much attention [10].

*Zingiber Officinale Roscoe* is the scientific name for ginger, which belongs to the Zingiberaceae family [11]. This spice has been used in Chinese and ayurvedic medicine for centuries [12]. The antioxidant properties of medicinal herbs are related to environmental conditions, weather, seasonal changes, geographical area, degree of ripe, growth, and many other factors during planting and harvesting [13]. The smell of fresh ginger is due to the presence of a group of phenolic compounds called gingerol, similarly, the smell of dried ginger is due to the presence of shogaols, which are dehydrated compounds of gingerols. Ginger has been declared to be safe by the US food and drug administration [14]. It has beneficial features due to bioactive compounds like gingerol, shogaol, paradol, and zingerone [15].

Although several animal studies have been conducted to assess the impact of ginger on metabolic indicators in DKD, a systematic review has not been initiated in association with this matter. Several systematic reviews showed the potential effects of ginger supplementation on glycemic control, lipid profile, inflammatory markers, and oxidative stress in patients with diabetes, hyperlipidemia, arthritis, neurological diseases, asthma, and stroke disease [16–20]. Some studies found that ginger intake could significantly increase fasting blood glucose (FBS), total cholesterol (TC), triglycerides (TG), urea, creatinine (Cr), and urine protein or no significant change in FBS, urea, and Cr levels [21–24]. The inconsistent results obtained in different studies could be attributed to various factors such as ginger form, dose, and duration of intervention. A systematic review is required to comprehensively integrate results from studies. The goal of the present systematic review is to investigate the literature on ginger’s influence on glycemic indices, dyslipidemia, inflammatory markers, oxidative stress markers, renal function, and structure. The mechanisms of the impact of ginger are presented in the discussion.

## Methods

### Search strategy

PubMed, Scopus, Embase, ProQuest, and Google Scholar were used as search engines, and keywords were chosen from MeSH and non-MeSH terms including: (“Ginger” OR “Zingiber” OR “Shogaols” OR “zingerone” OR “Gingerols”) AND (“kidney” OR “renal” OR “dialysis” OR “Hemodialysis” OR “ End Stage Renal Disease” OR “ESRD” OR “chronic kidney disease” OR “CKD” OR “acute renal failure” OR “ARF” OR “nephropathy” OR “diabetic nephropathy” OR “Glomerular Filtration Rate” OR “GFR” OR “Albuminuria” OR “Proteinuria” OR “Creatinine”) AND (“diabetes” OR “diabetes mellitus” OR “type 2 diabetes” OR “T2DM” OR “type 1 diabetes” OR “T1DM” OR “gestational diabetes mellitus” OR “GDM” OR “ Insulin Dependent Diabetes Mellitus” OR “IDDM” OR “Non-Inslin Dependent Diabetes Mellitus” OR “NIDDM” OR “fasting blood sugar” OR “fasting blood glucose” OR “glucose intolerance” OR “glucose tolerant”(. Preferred reporting items for systematic reviews (PRISMA) guidelines were followed when conducting this review.

### Eligibility criteria

Studies on the effect of ginger supplementation on DKD were included in this study. The PICO strategy for the research question of the study was patient/ population (P): animals mice or rats); Intervention (I): supplementation with ginger; Comparison (C): placebo group; and outcome (O): changed glycemic indices, lipid profile, inflammatory markers, oxidative stress, and renal function indicator.

Included studies include animal studies, English-language journals, and studies examining the effects of ginger on DKD. Excluded studies include studies in which ginger is supplemented in combination with other substances, studies in which we did not have access to the full text, and studies in vitro.

### Data extraction

The first and third authors (PV and HR) screened the titles and abstracts of the qualifying studies separately. The relevant data including the first author’s name, year of publication, country, study population, sample size, gender of subjects, ginger dosage, duration of intervention, diabetes induction method, and outcome data were extracted. Eligible papers were assessed based on the goal checklist, the question of the study, and inclusion/ exclusion criteria. Articles not meeting the criteria for data collection were eliminated. Any discrepancies among reviewers were resolved through consultation with the authors. The quality of the selected studies was evaluated via a first author. Quality assessment studies used the syrcle’s tool.

### Quality assessment

To assess the quality of studies the SYRCLE’s RoB tool evaluated studies based on ten criteria: random allocation sequence, animals similar at baseline, allocation concealment, random housing, blinded investigators, random outcome assessment, blinded outcome collection, incomplete data justification, unbiased conclusions and other. Each study could ultimately have a total score of 10 points.

## Results

### Selected articles

**Fig. 1:**
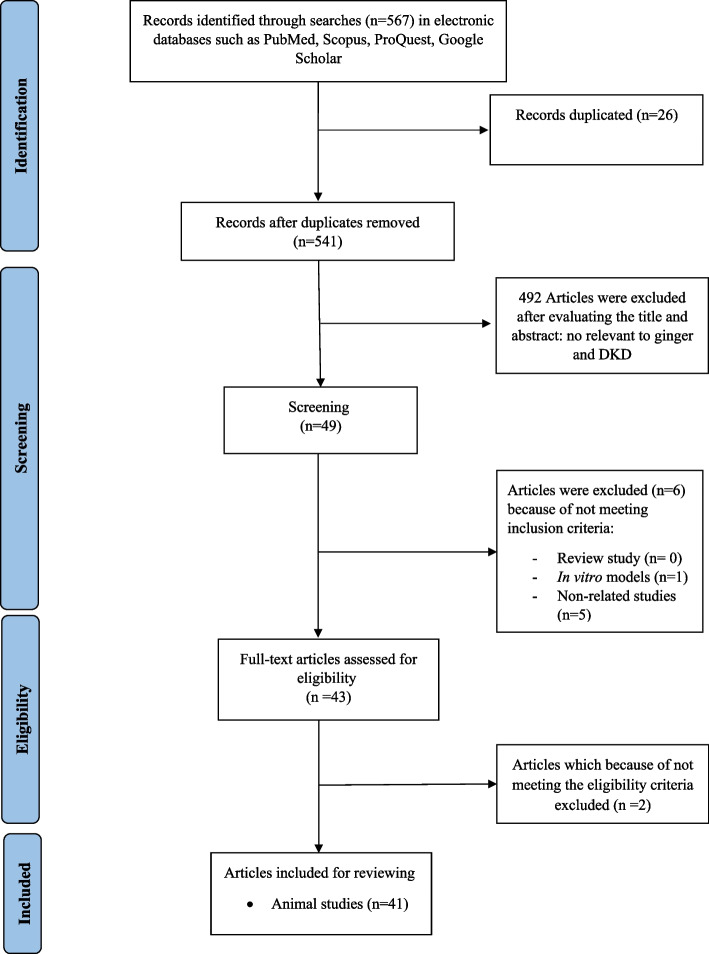
Flowchart of the process for literature review and study selection

Figure 1 depicts a flowchart of the research selection. The initial search resulted in a total of 567 articles, resulting in 541 non-duplicated publications after removing 26 articles. Following a review of titles and abstracts, 492 articles were eliminated. 6 studies were excluded due to the lack of inclusion criteria. Finally, the present review found 41 articles that meet the eligibility criteria. Table 1 summarizes the characteristics of chosen studies.

**Table 1:** characteristics of the included studies

Authors/date	Source	Models	sex	Sample size Int/ctrl	Substance induced diabetes	Daily dose	Ginger form	Duration	Results
Yi J-K et al. 2019 [25]	South Korea	C57BL/6J mice	M	6/3	STZ	5, 10 mg/kg	6-shogaol	2 weeks	- ↓ histopathological change in kidney - ↓ blood glucose
Rehman MU et al. 2019 [26]	India	Albino wister rats	M	6/6	STZ/ HFD	50, 100 mg/kg	Zingerone	16 weeks	- ↓ kidney damage - ↓ HbA1c - ↓ ROS - ↑ GSH, GPx, GR, SOD, and CAT - ↓ TC, LDL-C, TG and ↑ HDL-C - ↓TNF-a, IL-6, IL-1β and NF-κB - ↓ BUN, Cr
Xu Y et al. 2018 [27]	China	C57BL/KsJ db/db obese mice	M	10/10	db/db	25, 50 mg/kg	6-shogaol	12 weeks	- ↓ FBS, insulin, C peptide, and HbA1c - ↓ BUN, Cr, urinary albumin - ↓ TG and TC - ↓ pathological injuries of kidneys - ↓ TNFɑ, IL-6 and NFκB - ↓ GSH
Irshad F et al. 2018 [28]	Pakistan	Albino wister rats	M	15/15	Alloxan	200 mg/kg	aqueous extract	5 weeks	- ↓ Cr - ↓ kidney weight
Cui Y et al. 2018 [21]	China	C57BL/KsJ db/db obese mice	M	15/15	db/db	50 mg/kg	Zingerone	10 weeks	- ↓ insulin, C peptide, and HbA1c - ↔FBS - ↓ BUN, Cr, urinary albumin - ↓ TG and TC - ↓ TNF-ɑ and IL-6 - ↓ MDA - ↑ GSH - ↓ ROS - ↓ renal pathological change - ↓ kidney weight
Al Hroob AM et al. 2018 [29]	Egypt	Albino wistar rats	M	6/6	STZ	400, 800 mg/kg	ethanolic extract	6 weeks	- ↓ FBS and HbA1C - ↓ BUN, Cr, urea, and urine albumin - ↓ kidney damage - ↓ MDA - ↑ GSH, SOD, and CAT - ↓ TNF-α, IL-1β, and IL-6 - ↓ TG, TC, and LDL-C and ↑ HDL-C
Abdulsalam K et al. 2016 [22]	Saudi Arabia	Albino wister rats		10/10	NR	(0.5 − 2%) of ginger freeze-dried or extract	zingerone	6 weeks	- ↔ BUN, uric acid and urea - ↑ Cr - ↑ blood glucose
Kazeem MI et al. 2015 [24]	Nigeria	Albino Wistar rats	M	8/8	STZ	1.0 mL 500 mg/kg free and bound polyphenol	Acetone extracts	42 days	- ↓ urea - ↔ Cr - ↓ blood glucose - ↑ insulin - ↓ pathological change in kidney
Hanna ET et al. 2014 [30]	Egypt	Albino wister rats	M	7/7	Alloxan	0.5%, 1%, 5%	powder	6 weeks	- ↓ blood glucose - ↓TC and TG, LDL-C and ↑ HDL-C - ↓Cr and urea
Hajhosieni L et al. 2014 [31]	Iran	Albino wister rats	M	10/10	STZ	100 mg/kg	powder	8 weeks	- ↓MDA - ↑ TAC - ↓ kidney damage
Khaki A et al. 2010 [32]	Iran	Albino wister rats	M	10/10	STZ	100 mg/kg/day	powder	30 days	- ↓ pathological change in kidney - ↓ MDA - ↑TAC
Afshari AT et al. 2006 [33]	Iran	Albino wister rats	M	8/8	STZ	5% of daily food intake	powder	8 weeks.	- ↓ MDA - ↑ TAC - ↓ renal nephropathy
Al-Amin ZM et al. 2006 [34]	Kuwait	Sprague Dawley rats	M	8/8	STZ	500 mg/kg	Aqueous extract	7 weeks.	- ↓ blood glucose - ↓ TC, TG - ↓ urine protein
Al-Attar AM et al. 2007 [23]	Saudi Arabia	Albino wister rats	M	10/10	STZ	5% and 2.5% of diet	oil	2 weeks	- ↑blood glucose, TG, TC, LDL-C, and ↓ HDL-C - ↑ urine protein, urea, uric acid - ↓ Cr
Elshater A-EA et al. 2009 [35]	Egypt	Albino wister rats	M	10/10	Alloxan	4ml/kg	Aqueous extract	6 weeks	- ↓ blood glucose - ↓ TC, TG, LDL-C and ↑ HDL-C - ↓ Cr, urea, uric acid
Shanmugam K et al. 2009 [36]	India	Albino wistar rats	M	6/6	STZ	200 mg/kg	Ethanolic extract	30 days	- ↓ blood glucose
El-kott AF et al. 2010 [37]	Egypt	Albino wister rats	M	8/8	Alloxan	400 mg/kg	powder	4 weeks	- ↓ blood glucose - ↑ insulin - ↓ BUN - ↔ Cr, uric acid - ↓ pathological change in kidney
Kadah M 2010 [38]	Egypt	Albino wistar rats	M	8/8	STZ	2.5 g, 0.9%	Powder or oil	8 weeks	- ↓ blood glucose - ↑ insulin - ↓ TC, TG, LDL-C and ↑ HDL-C - ↓ urea, Cr - ↑ GPx and GSH - ↓ histopathological change in kidney
Ramudu SK et al. 2011 [39]	India	Albino wistar rats	M	6/6	STZ	200 mg/kg	Ethanolic extract	30 days	- ↓ blood glucose - ↓ renal tissue injuries
Abdulrazaq NB et al. 2012 [40]	Malaysia	Sprague-dawley rats	M	NR	STZ	100, 300, 500 mg/kg	Aqueous extract	30 days	- ↓ blood glucose - ↓ kidney weight
Ramudu SK et al. 2011 [41]	India	Albino wister rats	M	6/6	STZ	100, 200 mg/kg	Ethanolic extract	30 days	- ↓ blood glucose - ↓ MDA - ↓ TC, TG - ↓ pathological changes in kidney tissue
Shanmugam KR et al. 2011 [42]	India	Albino wister rats	M	6/6	STZ	1% and 2% of the diet	powder	30 days	- ↓ blood glucose - ↑ SOD, CAT, GPX, GR, and GSH - ↓ MDA - ↓ pathological changes in kidney
Eleazu C et al. 2013 [43]	Nigeria	Albino wister rats	M	6/6	STZ	10% of food intake	powder	3 weeks	- ↓ blood glucose - ↓ urinary protein - ↔ kidney weight
Sangi S et al. 2018 [44]	Saudi Arabia	Albino wister rats	M	5/5	STZ	1000 mg/kg	Aqueous extract	3 weeks	- ↓ blood glucose - ↓ urea, creatinine - ↓ TG, TC and HDL-C
Sangi S et al. 2017 [45]	Saudi Arabia	Albino wister rats	M	5/5	STZ	6% of diet	powder	8 weeks	- ↓ pathological change of kidney
Irshad F et al. 2018 [46]	Pakistan	Albino wister rats	M	15/15	Alloxan	200 mg/kg	Aqueous extract	5 weeks	- ↓ kidney damage
Abd Elwahab AH et al. 2015 [47]	Egypt	Albino wister rats	M	10/10	Alloxan	300 mg/kg of	Ethanolic extract	4 weeks	- ↓ blood glucose - ↓ urea, Cr and urine albumin - ↓ kidney damage - ↑ GSH - ↓ TNFα
Hassan DR et al. 2017 [48]	Egypt	Albino wister rats	M	7/7	STZ	125, 250, 500 mg / 100 g of diet	powder	4 weeks	- ↓ blood glucose - ↓ MDA - ↑ GSH - ↓ urea, Cr, uric acid
Jiyil M et al. 2019 [49]	India	Albino wister rats	M	5/5	STZ	400 mg/kg	Aqueous extract	21 days	- ↓ blood glucose - ↓ TC, TG and ↑ HDL-C - ↓ urea, uric acid, Cr
Al-Qudah MM et al. 2018 [50]	Jordan	Albino wister rats	F	5/5	Alloxan	500 mg/kg	Aqueous extract	21 days	- ↓ histopathological change in kidney
Kumari P et al. 2020 [51]	India	mice	NR	NR	Alloxan	80 mg/kg	Aqueous extract	16 weeks	- ↓ urea, uric acid - ↓ Blood glucose - ↓ pathological change in kidney
Almatroodi SA et al. 2021 [52]	Saudi Arabia	Albino wistar rats	M	8/8	STZ	10 mg/kg	6-gingerol	8 weeks	- ↓ FBS - ↓ TC, TG and LDL-C - ↓ urea, Cr - ↓ MDA - ↑ GSH, CAT, SOD - ↓ TNFα, IL-6, IL-1β - ↓ kidney damage
payami S-A et al. 2018 [53]	Iran	Albino wister rats	M	4/4	STZ	200, 400 mg/kg	Hydroalcoholic extract	8 weeks	- ↓ Blood glucose - ↓ urinary protein and Cr - ↓ histopathological change in kidney
Taha AM et al. 2020 [54]	Egypt	Albino wister rats	M	10/10	STZ	500 mg/kg	powder	6 weeks	- ↓ LDL-C and TC - ↓ urea
Irshad F et al. 2018 [55]	Pakistan	Albino wister rats	M	15/15	Alloxan	200 mg/kg	Aqueous extract	5 weeks	- ↓ Blood glucose - ↓ Histopathological change in kidney
Johti M et al. 2016 [56]	India	Albino wister rats	M	6/6	STZ	10 mg/kg	Zingerone	30 days	- ↓ Blood glucose - ↓ TC, TG, LDL-C and ↑ HDL-C - ↓ Histopathological change in kidney
Thomson M et al. 2013 [57]	Kuwait	Sprague –dawley rats	M	14/10	STZ	500 mg/kg	Aqueous extract	8 weeks	- ↓ Blood glucose - ↓Cr, uric acid - ↓ Urine protein - ↑ insulin
Al-Qattan KK et al. 2007 [58]	Kuwait	Sprague¬ dawley rats	M	10/10	STZ	500 mg/kg	extract	7 weeks	- ↓ Blood glucose - ↓ Histopathological change in kidney - ↓ Urine protein
Yassin S et al. 2019 [59]	Egypt	Sprague¬ dawley albino rats	M	8/8	STZ	200 mg/kg	Ethanolic extract	42 days	- ↓ Histopathological change - ↓ Uric acid, BUN and Cr - ↓ Blood sugar, HbA1c and ↑ Insulin - ↑ GPx, SOD and CAT - ↓ TC, TG, LDL-C and ↑ HDL-C
Ghudhaib KK. 2018 [60]	Iraq	Mice	NA	10/10	Alloxan	50, 100 mg/ml	Ethanolic extract	30 days	- ↓ TC, TG and ↑HDL-C - ↔ Cr, uric acid and ↓ Urea - ↓ Blood sugar and ↑insulin - ↑TAC
Al Malki WH et al. 2018 [61]	Saudi Arabia	Albino wister rats	M	20/20	STZ/ HFD	NR	6- shogaol	16 weeks	- ↓ Blood glucose - ↓ BUN, Cr and urine protein - ↓ NFκB, TNFα - ↓ Renal damage

*Ctrl* control, *Int* Intervention, *M* Male, *F* Female, *NR* not reported, *FBS* Fasting blood sugar, *TC* total cholesterol, *TG* triglycerides, *LDL-C* Low-Density lipoprotein-cholesterol, *HDL* High-density lipoprotein-cholestrol, *Cr* Creatinine, *ROS* Reactive oxygen species, *SOD* Superoxide dismutase, *CAT* catalase, *GPx* glutathione peroxidase, *GSH* Glutathione, *BUN* Blood urea nitrogen, *MDA* Malondialdehyde, *TAC* total antioxidant capacity, *IL6* Interleukin6, *STZ* streptozotocin, *HFD* High-fat diet, *TNFα* Tumor necrosis factor α, *HbA1c* hemoglobin A1c, ↓ decrease; ↑ increase; ↔ not changed

### Characteristics of the included studies

In total, after screening and deleting duplicate articles, forty-one studies were selected for this systematic review. All studies assessed diabetic mice or rats. Ginger was used in different shapes in this study, including ginger powder, ginger oil, aqueous ginger extract, ethanolic ginger extract, and bioactive compounds such as zingerone and shogaol. Ginger and ginger extract treatment dosages ranged from 80 to 1000 mg/kg and bioactive compounds treatment dosages ranged from 5 to 100 mg/kg. Intervention duration ranged from 2 to 16 weeks. Location of studies performed as follows: 9 in Egypt [29, 30, 35, 37, 38, 47, 48, 54, 59], 8 in India [26, 36, 39, 41, 42, 49, 51, 56], 6 in Saudi Arabia[22, 23, 44, 45, 52, 61], 4 in Iran [31–33, 53], 3 in Kuwait [34, 57, 58], 3 in Pakistan [28, 46, 55], 2 in Nigeria [24, 43], 2 in China [21, 27], 1 in South Korea [25], Jordan [50], Iraq [60] and Malaysia [40]. Studies were done from 2006 to 2021.

### Quality assessment

**Fig. 2:**
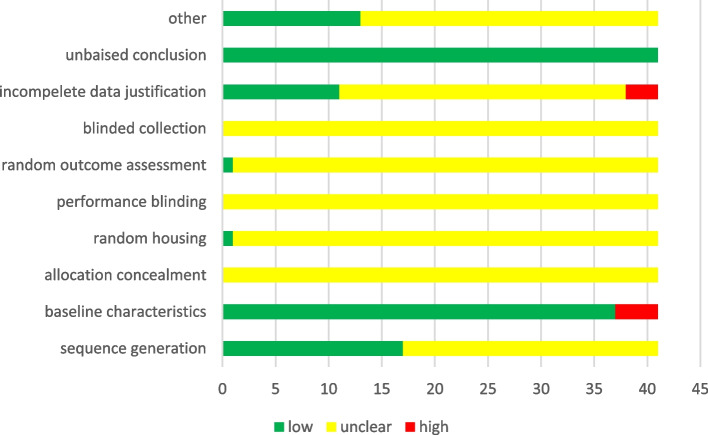
Results of the SYRCLE’s tool for assessing the risk of bias

A summary of the results of the quality assessment is demonstrated in Fig. 2. In the majority of studies, performance bias, detection bias, and allocation concealment were found to be unclear risks of bias.

#### Ginger and glycemic control in DKD

Twenty-eight of 31 studies showed that ginger intake lowers blood glucose levels [24, 25, 27, 29, 30, 34–43, 47–49, 51–53, 55–61]. On the contrary, in 2 studies, blood glucose levels increased [22, 23]. One study did not show any meaningful changes [21]. 6 out of 7 studies reported that ginger increases serum insulin levels [24, 37, 38, 57, 59, 60], whereas in another study the result was reversed [27]. Ginger reduced hemoglobin A1c (HbA1c) and C peptide in all studies that examined these biomarkers [21, 26, 27, 29, 59]. Finally, to assess the impact of ginger consumption, a subgroup analysis was performed for ginger forms. In the bioactive compound subgroup of ginger, 6-shogaol had a better effect on blood sugar than zingerone. In the ginger powder subgroup, hypoglycemia’s effect increases with increasing dose intake. In the ginger extract subgroup with a dose of 500 mg/kg or less, hypoglycemia increases with increasing dose. However, by a dose of more than 500 mg/kg of ginger extract, hypoglycemia was reduced.

#### Ginger and dyslipidemia in DKD

Sixteen out of 41 articles examined the effect of ginger on the lipid profile. The reduction of TC and TG were also reported by 15 [21, 26, 29, 30, 34, 35, 38, 41, 44, 49, 52, 54, 56, 59, 60] and 14 [21, 26, 29, 30, 34, 35, 38, 41, 44, 49, 52, 56, 59, 60] studies respectively. Ginger has been shown to improve low-density lipoprotein-cholesterol (LDL-C) [26, 29, 30, 35, 38, 52, 54, 56, 59] and high-density lipoprotein-cholesterol (HDL-C) [26, 29, 30, 35, 38, 49, 56, 59, 60] levels in nine studies. One study reported contradictory results for TC, TG, and LDL-C, as well as two studies for HDL-C [23, 44]. In the subgroup of bioactive compounds of ginger, by increasing the received dose, improving dyslipidemia increased. In the subgroup of ginger extract, higher doses had a better effect on TC, TG, LDL-C, and HDL-C levels. However, with a dose of more than 800 mg/kg of ginger extract, HDL-C was reduced.

#### Ginger and oxidative stress indices in DKD

Ginger reduced the malondialdehyde (MDA) levels in all 9 studies that examined it [21, 29, 31–33, 41, 42, 48, 52]. In all studies, the impact of ginger on the antioxidant defense system was evaluated, and positive results were found. In all studies, ginger elevated the level of glutathione (GSH) [21, 26, 27, 29, 38, 42, 48, 49, 52], catalase [26, 29, 42, 52, 59], superoxide dismutase (SOD) [26, 29, 42, 52, 59], glutathione reductase (GR) [26, 42], glutathione peroxidase (GPx) [26, 38, 42, 59] and total antioxidant capacity (TAC) [31–33, 60] factors. Similarly, 2 studies reported that administration of ginger decreased reactive oxygen species (ROS) levels [21, 26]. In the subgroup of the bioactive compounds of ginger, higher doses had a better effect on GSH levels. In the subgroup of ginger powder, higher doses had a better effect on MDA levels.

#### Ginger and inflammation biomarkers in DKD

Seven out of 41 studies investigated the influence of ginger on inflammatory markers. Ginger diminished tumor necrosis factorα (TNFα), interleukin6 (IL6), interleukin1β (IL1β), and nuclear factor kappa-light chain-enhancer of activated B cells (NFκB) serum levels in 7 [21, 26, 27, 29, 47, 52, 61], 5 [21, 26, 27, 29, 52], 3 [26, 29, 52], and 3 [26, 27, 61] studies, respectively. The studies did not show any adverse effects. Higher doses had a better effect on TNFα and IL6 levels in the subgroup of the bioactive compounds of ginger.

#### Ginger and renal function in DKD

Twenty-four studies evaluated the potential effect of ginger on kidney function indicators. In 17 of 21 studies ginger supplementation reduced serum creatinine levels [21, 23, 26–30, 35, 38, 44, 47–49, 52, 53, 57, 59]. Creatinine levels, on the other hand, increased in one study [22] and remained unchanged in three others [24, 37, 60]. Ginger decreased serum levels of urea, blood urea nitrogen (BUN), and uric acid in 13 [24, 29, 30, 35, 38, 44, 47–49, 51, 52, 54, 60], 6 [21, 26, 27, 29, 37, 59], and 6 [35, 48, 49, 51, 57, 59] studies, respectively. On the contrary, urea and uric acid levels were increased in one study [23]. Uric acid levels were not significantly changed in three studies [22, 37, 60]. One study showed no meaningful changes in urea and BUN levels [22]. Higher doses had a better effect on BUN and Cr levels in the subgroup of the bioactive compounds of ginger. By increasing ginger extract intake, the effect on uric acid increased. In the subgroup of ginger powder, higher doses had a better effect on urea levels.

#### Ginger and proteinuria in DKD

Urinary protein was decreased in 6 studies [34, 43, 53, 57, 58, 61] and increased in one study [23]. Moreover, urine albumin was decreased in 4 studies [21, 27, 29, 47].

#### Ginger and changes in histomorphology and structural renal in DKD

Among the studies, twenty-six evaluated the influence of ginger on histopathological changes in kidneys [21, 24–27, 29, 31–33, 37–39, 41, 42, 45–47, 50–53, 55, 56, 58, 59, 61]. All studies examining histomorphological changes showed beneficial effects. The beneficial impacts of ginger on bowman’s capsule atrophy, the surface area of bowman’s capsule, and bowman’s space were demonstrated in seven studies [21, 24, 25, 27, 33, 45, 46]. In 8 articles, necrosis of tubular and glomerular cells was reduced [21, 27, 29, 39, 41, 42, 58, 59], also hajhosseini et al. found that the number of apoptotic cells was reduced [31]. In 12 studies ginger reduced dilation and degeneration of tubules [21, 25, 27, 29, 32, 33, 39, 41, 42, 47, 59, 61]. Additionally, in four studies, the weight of the kidneys decreased at the end [21, 27, 28, 40], although, in one study, the weight of the kidneys did not change significantly [43].

## Discussion

The present systematic review was conducted to discover the impact of different forms of ginger on metabolic indicators in DKD. The findings, to the best of our knowledge, show some positive effects of ginger in DKD. The result of the current systematic review exhibited that ginger has a beneficial effect on blood levels of glucose, insulin, C-peptide, and HbA1C. However, the results on blood glucose in the studies done by Abdulsalam et al. and Al-Attar et al. were contradictory [22, 23]. The conflicting results seem to be due to the fact that the dose of ginger was not clear because it was expressed as a percentage of the diet. However, in the studies with positive results, ginger was prescribed in mg/kg with specified dosages. Also, Xu Y et al. showed that 25 or 50 mg/kg of 6-shogaol reduced insulin serum levels [27]. Notably, in this study 6-shogaol was used, while other studies were based on ginger supplementation. There was insufficient evidence to draw conclusions about homeostatic model assessment of insulin resistance (HOMA-IR).

**Fig. 3:**
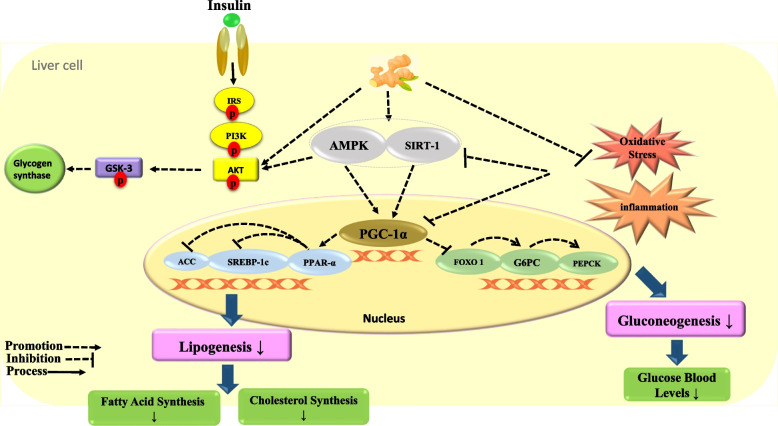
The possible effect of zingiber officinale on gluconeogenesis and lipogenesis in the hepatocyte cell. Ginger reduces hepatic cholesterol production and blood sugar levels via the AMPK pathway

Several possible mechanisms have been suggested for ginger’s effect on glycemic indices. A mechanism was expressed in Fig. 3 to explain how ginger can improve blood glucose levels in liver cells. Ginger activates the AMP-activated protein kinase (AMPK) pathway [62]. Activation of this pathway inhibits forkhead box protein O1 (FOXO1), an important transcription factor in regulating the expression of genes involved in hepatic glucose production (gluconeogenesis) such as phosphoenolpyruvate carboxykinase PEPCK and glucose-6- phosphatase (G6pase), resulting in decreased hepatic glucose production [63]. Also, ginger inhibits the hepatic phosphorylase enzyme activity and suppresses glycogenolysis in liver cells, while increases the activity of glycogenesis enzymes [64]. According to a study, ginger can also increase the activity of hepatic glycolytic enzymes such as glucokinase, phosphofructokinase, and pyruvate kinase [29]. Another suggested mechanism is inhibition of the hepatic glucose 6 phosphatase enzyme activity, thereby reducing the conversion of glucose 6 phosphates to glucose, causes to decreasing blood glucose levels [65].

**Fig. 4:**
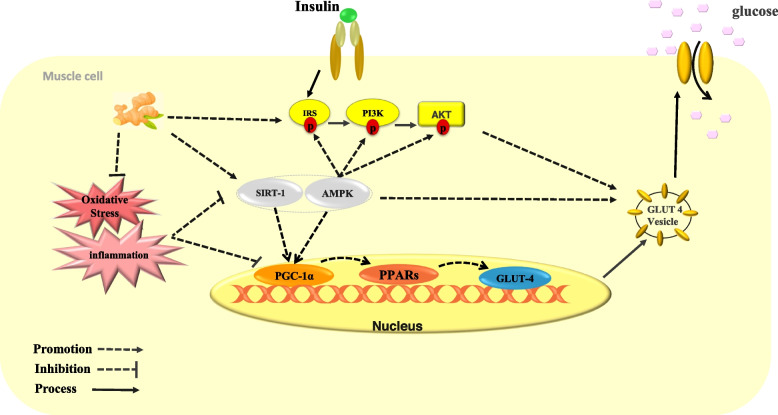
The possible effect of ginger on insulin sensitivity in the skeletal muscle cells. Ginger increases the expression of the GLUT4 gene and the translocation of GLUT4 to the plasma membrane of muscle cells via the AMPK pathway, which leads to enhanced glucose uptake

In one study, glucose uptake in rat muscle cells was increased due to translocation of glucose transporter4 (GLUT4) transporter to the plasma membrane, and the rise in GLUT 4 gene expression facilitated insulin-independent glucose uptake [66]. In addition, ginger activates the AMPK pathway [62]. Activation of AMPK by increasing the phosphorylation of insulin receptor substrate (IRS), phosphoinositide 3-kinase (PI3K), and protein kinase B (Akt) tyrosine roots improves insulin signaling and increases the translocation of GLUT4 transporter to the plasma membrane surface, and increases the entry of glucose into the cell [67]. Figure 4 shows how ginger may affect insulin sensitivity. Moreover, ginger can reduce Insulin resistance in skeletal muscle [68].

Dyslipidemia is one of the predictors of DKD progression [7, 69, 70]. In general, based on the present study, the lipid profile was improved due to ginger supplementation. Although the results of Attar’s study on lipid profile were quite the opposite, so that TC, TG, and LDL-C were increased and HDL-C was decreased. In Attar’s study, the dose of ginger oil was 2.5 and 5% of the diet and supplementation lasted for 2 weeks [23]. Also, Sangi S et al. showed that the application of 1000 mg/kg ginger aqueous extract for 3 weeks reduced serum HDL-C [44]. These results appear to be inconsistent due to the short duration of supplementation.

As shown in Fig. 3, ginger increases the expression of the peroxisome proliferator-activated receptor alpha (PPAR-α) gene by activating the AMPK-SIRT-PGC-1α pathway in the liver, which leads to the inhibition of the expression of regulatory genes such as sterol regulatory element binding protein1c (SREBP-1c) and acetyl-CoA carboxylase (ACC) in lipogenesis. As a result of the expression of ACC and SREBP1C genes, the synthesis of fatty acids and cholesterol is reduced [68]. Some other possible mechanisms were proposed for lowering lipid levels with ginger intake in two systematic reviews [20, 71]: (1) The reduction of the cholesterol biosynthesis by reducing farnesyl diphosphate liver production, (2) Induction of the conversion of cholesterol into bile acids and increased cholesterol excretion, (3) The liver uptake LDL-C from blood circulation and reduces cholesterol synthesis, (4) Increased pancreatic lipase, (5) Inhibition of lipid hydwrolysis in the intestine, (6) PPARδ pathway activation, (7) Decreased retinol-binding protein (RBP) expression, which is an indicator of hyperlipidemia, (8) The presence of niacin in ginger, which reduces TG and VLDL-C and uptake of LDL-C by liver, (9) The reduction of the conversion of excess carbohydrate to TG by reducing the expression of carbohydrate response element-binding protein (ChREBP) gene.

**Fig. 5:**
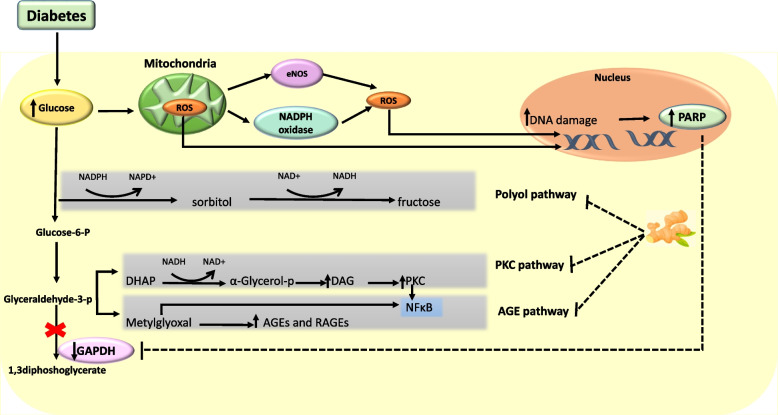
Schematic representation of possible mechanisms of the effect of ginger on oxidative stress

Inflammation and oxidative stress play an important role in the pathogenesis and progression of DKD [72, 73]. The results of the current systematic review support the beneficial effect of ginger on both inflammation and oxidative stress. Possible mechanisms for reducing inflammation by ginger are as follows: (1) NF-κB signaling pathway suppression [68], (2) Inhibition of cyclooxygenase2 (COX-2) and lipoxygenase, thus suppressing arachidonic acid (AA) metabolism, (3) Inhibition of prostaglandin synthesis, (4) Presence of some compounds in ginger that are serotonin blockers and reduce inflammation and prostaglandins production [17]. Hyperglycemia increases the production of reactive oxygen species )ROS(. Ginger reduces ROS directly or indirectly by lowering blood glucose [29]. Also ginger reduces oxidative stress and lipid peroxidation by scavenging free radicals [74]. Figure 5 shows the possible mechanisms for reducing oxidative stress, which are as follows: 1) preventing the formation of advanced glycation end products (AGEs) via nuclear factor erythroid 2-related factor2 (Nrf2) dependent pathway [3]. 2) inhibition of protein kinase C [75]. 3) inhibition of polyol pathway [76].

Overall, renal function indicators were improved based on the obtained results of the present study. However, urea and uric acid levels in Al-Attar AM et al. study and creatinine levels in Abdulsalam K et al. study were increased [22, 23]. The inconsistent results seem to be due to the fact that the exact effective dosage of ginger was not clear because in most studies, it was expressed as a percentage of the diet. As a matter of fact, in the studies showing the beneficial effects of ginger, it was prescribed in mg/kg with specified dosages.

Possible mechanisms for improving renal function by ginger are as follows: (1) Hyperglycemia induces free radicals that attribute to the activation of various downstream signaling cascades leading to structural and functional changes in the renal [77]. Ginger improves renal function through scavenging free radicals [74], (2) AGEs have a key role in the pathogenesis and the progression of DKD. AGEs accumulate in DKD as a result of decreased excretion and increased production resulting from oxidative stress [78]. Bioactive ginger components reduce protein glycation by trapping methylglyoxal [78]. Therefore, ginger inhibits the initiation and progression of DKD by reducing the glycation of proteins, (3) Urea induces free radical production and apoptosis that leads to functional changes in the kidney [79]. Ginger supplementation may reduce urea by inhibiting urea re-absorption in nephrons. Polyphenols and flavonoids present in ginger may play a role in renoprotective activities and lowering serum urea, creatinine, and uric acid levels [80], (4) In DKD lipid accumulation occurs in tubule epithelial cells, leading to kidney fibrosis. Xu Y et al. and Ramudu SK et al. showed that ginger reduced lipid content in kidney tissues [27, 41]. Therefore, ginger improves renal function and structure by reducing lipid accumulation.

Although proteinuria was decreased based on the current study, it was increased in the study done by Al-Attar AM et al. The ginger used in this study was in oil form, while in the other studies reviewed, powder, extract, or bioactive compounds of ginger were used, one of the probable reasons for different results. Notably, ginger reduces glomerular and tubular degeneration, reducing the thickening of glomerular basement membrane and restoring the integrity of kidney tissue membranes [26, 39, 52]. the possible mechanisms underlying the reduced proteinuria observed in the studies.

All studies that examined histomorphological changes of kidney showed beneficial effects of ginger. Several studies have shown that ginger improves pathological changes such as cytotoxicity caused by hyperglycemia [21], cell apoptosis [31], and bleeding in the cortical area of the kidney [32], repairs kidney damage, and restored membrane integrity in renal tissue and structural derangement [26].

### Human studies

Recently, a clinical trial was published that investigated the effect of ginger on renal function in patients with T2DM [81]. Elsaadany et al. reported that 3000 mg/day of ginger reduced Cr, but not changed BUN significantly. Also, reported that FBS was reduced, but the reduction of HbA1c was not significant. HbA1c measures usually show the average blood glucose over the past 2 to 3 months. As the study duration was eight weeks, it seems that the lack of significant decrease in HbA1c is due to the short duration of supplementation. Also, reported that TG was reduced, but TC, LDL-C, and HDL-C did not meaningful changes. In agreement with these results, Pourmasoumi et al. demonstrated that low dose of ginger (≤ 2000 mg/day) had greater lowering impact on TG and TC [20].

### Knowledge gaps and future direction

Due to the lack of human studies, future well-designed clinical trials with large sample sizes, various dosages, and long durations are required to reach definitive results about the use of ginger in the prevention and reduction of complications of DKD. Different dosages, supplementation duration, and diverse forms of ginger were the possible reasons for the inconsistency of the results. Not registering the study protocol was another study limitation.

## Conclusion

As a whole, the results of the present systematic review indicated that ginger may have several beneficial effects on glycaemic indices, oxidative stress, inflammatory markers, lipid profile, and some renal function indicators. Although the results seem promising, further human trials are required to achieve more informative and conclusive results.

## Data Availability

The data is available for review.
